# ﻿Molecular and morphological evidence supports the resurrection of *Chrysospleniumguangxiense* H.G.Ye & Gui C.Zhang (Saxifragaceae)

**DOI:** 10.3897/phytokeys.243.125742

**Published:** 2024-06-25

**Authors:** Long-Fei Fu, Tian-Ge Yang, De-Qing Lan, Xi-Tang Chen, Hong Liu

**Affiliations:** 1 Guangxi Key Laboratory of Plant Conservation and Restoration Ecology in Karst Terrain, Guangxi Institute of Botany, Guangxi Zhuang Autonomous Region and Chinese Academy of Sciences, Guilin 541006, China Guangxi Zhuang Autonomous Region and Chinese Academy of Sciences Guilin China; 2 Hubei Provincial Key Laboratory for Protection and Application of Special Plant Germplasm in Wuling Area of China, College of Life Sciences, South-Central Minzu University, Wuhan 430074, China South-Central Minzu University Wuhan China; 3 Hubei Jiugongshan National Nature Reserve Administration, Xianning 437625, China Hubei Jiugongshan National Nature Reserve Administration Xianning China

**Keywords:** *Chrysosplenium*, phylogeny, plastid genome, Saxifragaceae, taxonomy

## Abstract

*Chrysospleniumguangxiense* H.G.Ye & Gui C.Zhang was first described as a new species in 1994 but later synonymized in the Flora of China treatment with *C.glossophyllum* H.Hara. Plastid genomes and nrDNA sequences were used to infer the phylogenetic relationships of selected taxa in *Chrysosplenium*. Our phylogenetic analyses revealed that *C.guangxiense* belongs to sect. Alternifolia, is closely related to *Chrysospleniumhydrocotylifolium* H.Lév. & Vaniot but distant from *C.glossophyllum*. Morphologically, *C.guangxiense* could be easily distinguished from *C.glossophyllum* by having robust rhizomes, basal leaves with a long cuneate base and fewer teeth in the margin, curled sepal margins, and red, larger seeds. It could also be easily distinguished from *C.hydrocotylifolium* by possessing long elliptic leaves and a long cuneate leaf base. Along with the phylogenetic studies, the complete plastid genome of *C.guangxiense* was also reported. The plastid genome was 154,004 bp in length and comprised two inverted repeats (IRs) of 28,120 bp, separated by a large single-copy of 80,646 bp and a small single-copy of 17,118 bp. A total of 111 functional genes were discovered, comprising 78 protein-coding genes, 29 tRNA genes, and four rRNA genes. Based on assessment of morphological and molecular data *Chrysospleniumguangxiense* H.G.Ye & Gui C.Zhang is resurrected from *C.glossophyllum* H.Hara at species level. A global conservation assessment classifies *C.guangxiense* as Vulnerable (VU).

## ﻿Introduction

*Chrysosplenium* L. (Saxifragaceae) comprises more than 70 species of perennial herbs (Kim et al. 2019; Fu et al. 2020, 2021). *Chrysosplenium* is distributed throughout Asia, America and Europe (Pan and Ohba 2001; Soltis 2007). The latest checklist of Chinese *Chrysosplenium* included 35 species (Pan and Ohba 2001). Their earlier revisions classified the genus into two subgenera (subg. Chrysosplenium and subg. Gamosplenium) based on leaf arrangement (Pan 1986a, b). This character was also considered by Hara, who divided Chrysosplenium into two sections, namely sect. Alternifolia and sect. Oppositifolia (Hara 1957). The following molecular analyses (Soltis et al. 2001; Fu et al. 2021) demonstrated that these two subgenera/sections are monophyletic and sister to each other, further confirming that leaf arrangement is a good indicator of the relationships within the genus. However, a recent systematic study based on a complete chloroplast genome and nrDNA data challenged this relationship as their results recovered an additional clade composed of two species with alternate leaves (as members of sect. Alternifolia). The newly defined clade was recognized as a basal clade sister to the rest of the species of *Chrysosplenium* (Yang et al. 2023).

*Chrysospleniumguangxiense* H.G.Ye & Gui C.Zhang was first described as a new species in 1994 by having ovate-elliptic leaves, an acuminate apex, a cuneate base, fewer dentate margins, and a depressed sepal apex, enabling it to be distinguished from its similar species, *C.glossophyllum* H.Hara (Ye and Zhang 1994). Subsequently, it appeared as a synonym of the latter in *Flora of China* without additional explanation (Pan and Ohba 2001). We assumed that the authors considered these differences were insufficient to distinguish them. It is possible that the scarcity of *C.glossophyllum* species in China, with its only population in Sichuan Province, may have led to this misinterpretation. Molecular data, however, could provide a means to confirm the systematic position of morphological similarities and to evaluate the phylogenetic informativeness of morphological characters (Scotland et al. 2003).

In 2019, we conducted an extensive investigation in Tianlin County, Baise City, Guangxi, China, the type locality of *Chrysospleniumguangxiense*. We collected a plant of *Chrysosplenium*, which was then confirmed as *C.guangxiense*. Following a thorough literature survey (Hara 1957; Pan 1992; Pan and Ohba 2001; Liu et al. 2016; Wei 2018; Kim et al. 2019; Fu et al. 2020, 2021; Wei et al. 2022), along with the molecular evidence, it was confirmed that *C.guangxiense* is a different species from *C.glossophyllum*.

## ﻿Materials and methods

### ﻿Morphology observations and conservation assessments

All morphological characters were studied based on the material from field and herbarium specimens using a dissecting microscope (SMZ171, Motic, China). For seed morphology, we also undertook scanning electron microscope (SEM) observations; seeds were collected from the field and dried with silica gel. The pre-treatments, including impurity removal, air-drying, and gold-coating, were performed following Fu et al. (2020). Observations and photographs were taken under a Hitachi SU8010 scanning electron micrograph. At least 15 seeds were used to determine their size and ornamentation. A conservation assessment was undertaken following the IUCN (2019).

### ﻿Genomic DNA extraction, sequencing, plastid genome and nrDNA assembly and annotation

The genomic DNA was extracted using the modified CTAB method (Doyle and Doyle 1987). The short-insertion library (300 bp) was constructed and then sequenced to obtain 2×150 bp paired-end data using the Illumina NovaSeq platform at Majorbio Company (Shanghai, China). The raw data was filtered through Trimmomatic v. 0.39 (Bolger et al. 2014) to obtain clean data, and then the clean data were quality-controlled using FastQC v. 0.11.9 (Simon 2020). The complete plastid genome and nrDNA sequence were assembled using GetOrganelle v. 1.7.5 (Jin et al. 2020), and annotation was performed using CPGAVAS2 (Shi et al. 2019) and PGA (Qu et al. 2019).

### ﻿Phylogenetic analysis

To confirm the phylogenetic placement of *Chrysospleniumguangxiense*, we undertook phylogenetic studies using the chloroplast (CP) genomes and nrDNA sequences obtained in a previous study (Yang et al. 2023). Forty-seven species of *Chrysosplenium* as in-group, and two species from other genera in Saxifragaceae and *Iteachinensis* Hook. & Arn. from Iteaceae as an out-group were sampled. The species names and GenBank accession numbers are listed in Table 1.

**Table 1.** Species names and GenBank accession numbers of plastid genomes and nrDNA sequence used in this study (* newly generated sequences).

Species	Location	Voucher specimens	Herbarium	Plastid GenBank number	nrDNA GenBank number
*Chrysospleniumalbum* Maxim.	Nikkou-shi, Japan	HSN09815	HSN	OK336556	OP154009
*Chrysospleniumaureobracteatum* Y.I.Kim & Y.D.Kim	Gangwon Province, South Korea	KYI-2009032	(Kim et al. 2018)	MG878089	MK989509
*Chrysospleniumbiondianum* Engl.	Shanxi, China	HZ2017050107362	HSN	OK336542	OP154015
*Chrysospleniumcarnosum* Hook.f. et Thoms.	Sichuan, China	HSN013113	HSN	OK336564	OP154016
*Chrysospleniumdavidianum* Decne. ex Maxim.	Sichuan, China	HSN06442	HSN	OK336537	OP154017
*Chrysospleniumdelavayi* Franch.	Sangzhi, Hunan, China	SZ2016080907105	HSN	OK336539	OP154018
*Chrysospleniumdubium* J. Gayex DC.	Georgia	P03_WF11	(Folk et al. 2019)	–	OP154019
*Chrysospleniumechinus* Maxim.	Nikkou-shi, Japan	HSN09817	HSN	OK336557	OP154020
*Chrysospleniumfauriae* Franch.	Nikkou-shi, Japan	HSN09823	HSN	OK336561	OP154021
*Chrysospleniumflagelliferum* Fr. Schmidt.	Nikkou-shi, Japan	HSN09816	HSN	OK336541	OP154022
*Chrysospleniumforrestii* Diels	Nikkou-shi, Japan	HSN7797	HSN	OK336565	OP154024
*Chrysospleniumgiraldianum* Engl.	Sichuan, China	JZ2018042507981	HSN	OK336548	OP154025
*Chrysospleniumglossophyllum* H. Hara	Sichuan, China	QCS2017102608035	HSN	OK336544	OP154026
*Chrysospleniumgrayanum* Maxim.	Nikkou-shi, Japan	HSN09810	HSN	OK336555	OP154027
*Chrysospleniumgriffithii* Hook.f. et Thoms.	Shanxi, China	HSN7760	HSN	OK336547	OP154028
*Chrysospleniumguangxiense* H.G.Ye & Gui C.Zhang	Guangxi, China	HSN13356	HSN	OP093635 ^*^	OR941245 ^*^
*Chrysospleniumhenryi* Franch.	Sangzhi, Hunan, China	HSN7505	HSN	OK336532	OP154030
*Chrysospleniumhydrocotylifolium* H. Lév. & Vaniot	Hubei, China	HSN09188	HSN	OK336540	OP154031
*Chrysospleniumjaponicum* (Maxim.) Makino	Zhejiang, China	HSN7909	HSN	OK336554	OP154032
*Chrysospleniumkamtschaticum* Fisch. ex Seringe	Shimane-ken, Japan	DG2019032310004	HSN	MT371065	OP154033
*Chrysospleniumkiotense* Ohwi.	Nikkou-shi, Japan	HSN09818	HSN	OK336558	OP154034
*Chrysospleniumlanuginosum* Hook.f. et Thoms.	Anhui, China	BD2017030507343	HSN	OK336534	OP154035
*Chrysospleniumlectus*-*cochleae* Kitagawa	Jilin, China	HSN7379	HSN	OK336550	OP154036
*Chrysospleniummacrophyllum* Oliv.	Hubei, China	BD2017030507344	HSN	MK973001	OP154037
*Chrysospleniummacrospermum* Y.I.Kim & Y.D.Kim	Jilin, China	CBS2016062406656	HSN	OK336562	OP154038
*Chrysospleniummacrostemon* Maxim. ex Franch. et Sav.	Nikkou-shi, Japan	HSN09820	HSN	OK336560	OP154039
*Chrysospleniummicrospermum* Franch.	Jinfo Mountain, Chongqing, China	HSNTG025	HSN	OK336546	OP154040
*Chrysospleniumnepalense* D.Don	Tengchong, Yunnan, China	GLGH20170607375	HSN	OK336535	OP154043
*Chrysospleniumnudicaule* Bunge	Gansu, China	HSN07772	HSN	MZ424445	OP154044
*Chrysospleniumoppositifolium* L.	Wales, UK	BGN_RN_W	(Folk et al. 2019)	OR397749	OP154057
*Chrysospleniumpilosum* Maxim.	Nikkou-shi, Japan	HSN09819	HSN	OK336559	OP154045
*Chrysospleniumvaldepilosum* (Ohwi) S.H.Kang & J.W.Han	Jilin, China	HSN09819	HSN	OR397753	OP154046
*Chrysospleniumqinlingense* Z.P.Jien ex J.T.Pan	Sichuan, China	HSN7980	HSN	OK336549	OP154047
*Chrysospleniumramosum* Maxim.	Jilin, China	SJH2017052107372	HSN	MK973002	OP154048
*Chrysospleniumsedakowii* Turcz.	Irkutsk, Russia	P02_WC8	(Folk et al. 2019)	–	OP154049
*Chrysospleniumserreanum* Hand.-Mazz.	Jilin, China	SJH2017052107371	HSN	OK336538	OP154050
*Chrysospleniumsinicum* Maxim.	Hunan, China	TPS2017042407504	HSN	MT362051	OP154051
*Chrysospleniumtaibaishanense* J.T.Pan	Shanxi, China	HSN7761	HSN	OK336552	OP154052
*Chrysospleniumuniflorum* Maxim.	Tibet, China	HSN7380	HSN	OK336533	OP154053
*Chrysospleniumzhouzhiense* Hong Liu	Shanxi, China	HSN13356	HSN	OK336551	OP154055
*Chrysospleniumalternifolium* L.	Shimane-ken, Japan	DG2019032310003	HSN	OK336545	OP154010
*Chrysospleniumtetrandrum* (N. Lund) Th. Fries	Nunavut, Canada	Brysting_01-065_CAN	CAN	OR397750	OP154052
*Chrysospleniumwrightii* Franch. & Sav.	Yukon, Canada	Bennett_08-125_CAN	CAN	OR397751	OP154059
*Chrysospleniumvaldivicum* Hook.	Chile	P04_WG8	HSN	OR397752	OP154060
*Chrysospleniumzhangjiajieense* X.L.Yu, Hui Zhou & D.S.Zhou	Hunan, China	ZJ2016031506369	HSN	OK336563	OP154054
*Peltoboykiniatellimoides* (Maxim.) Hara	Henan, China	PT210814	(Yang et al. 2022)	MZ779205	JQ895246
*Saxifragastolonifera* Curt.	Hubei, China	S313	(Chen et al. 2022)	NC_037882	MK092506
*Iteachinensis* C.K.Schneider	Hunan, China	S371	–	NC_037884	MG730867

The chloroplast protein-coding genes (cpPCGs) were extracted from the CP genome using PhyloSuite v.1.2.3 (Zhang et al. 2020). These cpPCGs and nrDNA sequences were aligned by MAFFT v. 7.4 (Katoh and Standley 2013), and concatenated using PhyloSuite v.1.2.3 (Zhang et al. 2020) to form the cpPCGs+nrDNA matrix. The phylogenetic analyses of *Chrysosplenium* based on cpPCGs, nrDNA and cpPCGs+nrDNA matrices were performed using maximum likelihood (ML) and Bayesian inference (BI), respectively. The ML analyses were conducted using IQ-TREE v. 2.1.2 (Nguyen et al. 2015) with 1,000 bootstrap replicates and the default ModelFinder (Kalyaanamoorthy et al. 2017) to find GTR+F+I+G4 as the best-fit substitution model. Tree visualization was achieved in Figtree v. 1.4.3 (http://tree.bio.ed.ac.uk/software/figtree/). For BI analysis, MrBayes v. 3.2.6 (Ronquist et al. 2012) was employed to obtain a maximum clade credibility (MCC) tree. BI analysis was performed using one million generations, two runs, 25% trees discarded as burn-in, and trees sampled every 1,000 generations (1,000 trees sampled in total) with the GTR model.

## ﻿Results

### ﻿Characteristics of the complete chloroplast genome

**Figure 1.**
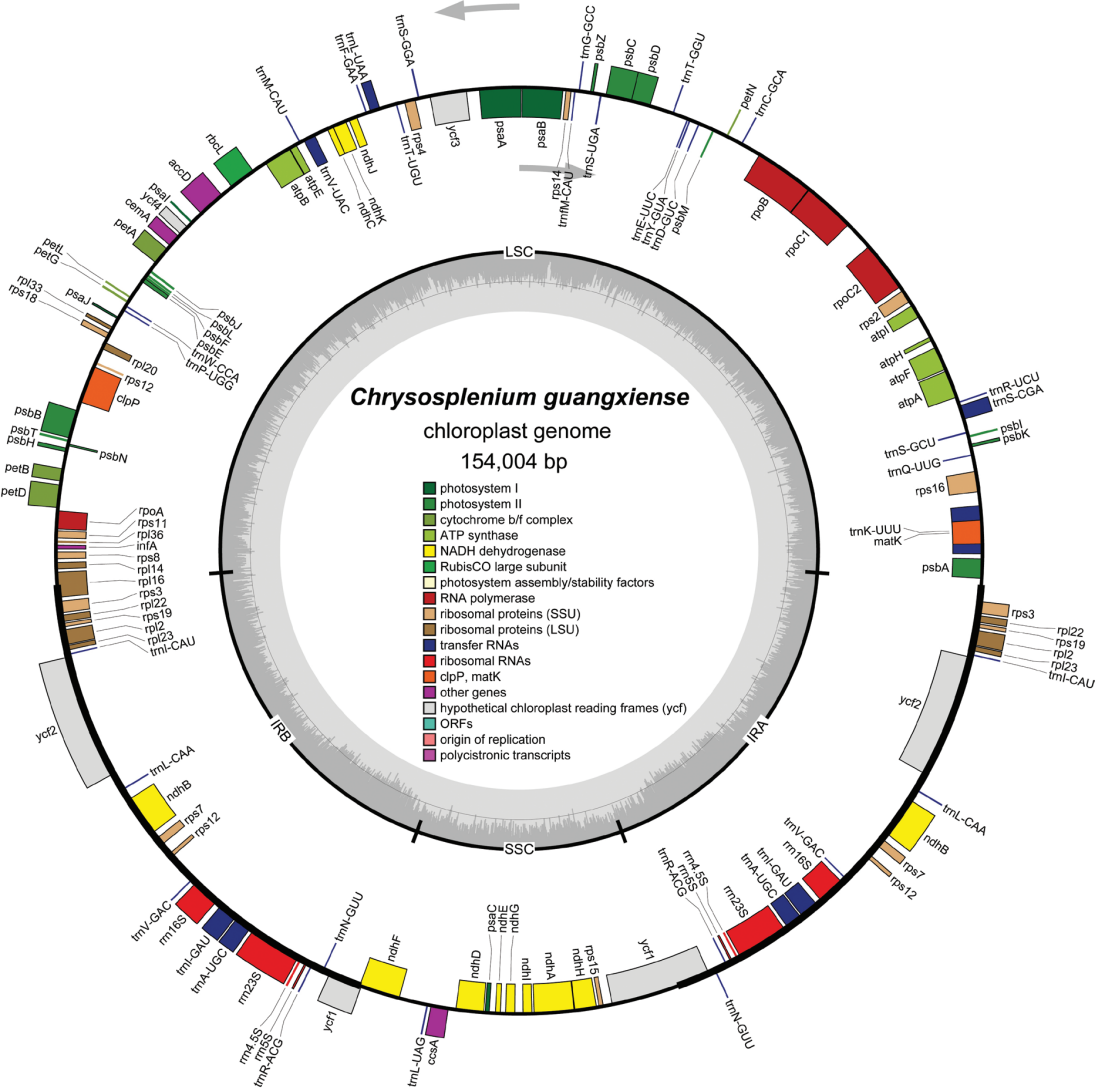
Plastid genome map of *Chrysospleniumguangxiense*. The thick lines on the outer complete circle identify the inverted repeat regions (IRa and IRb). The innermost track of the plastome shows the GC content. Genes on the outside and inside of the map are transcribed in clockwise and counter directions, respectively.

The CP genome of *Chrysospleniumguangxiense* comprised 154,004 bp (Fig. 1). The characteristics and statistics of the CP genome are summarized in Tables 4, 5.

### ﻿Molecular phylogenetic studies

**Figure 2.**
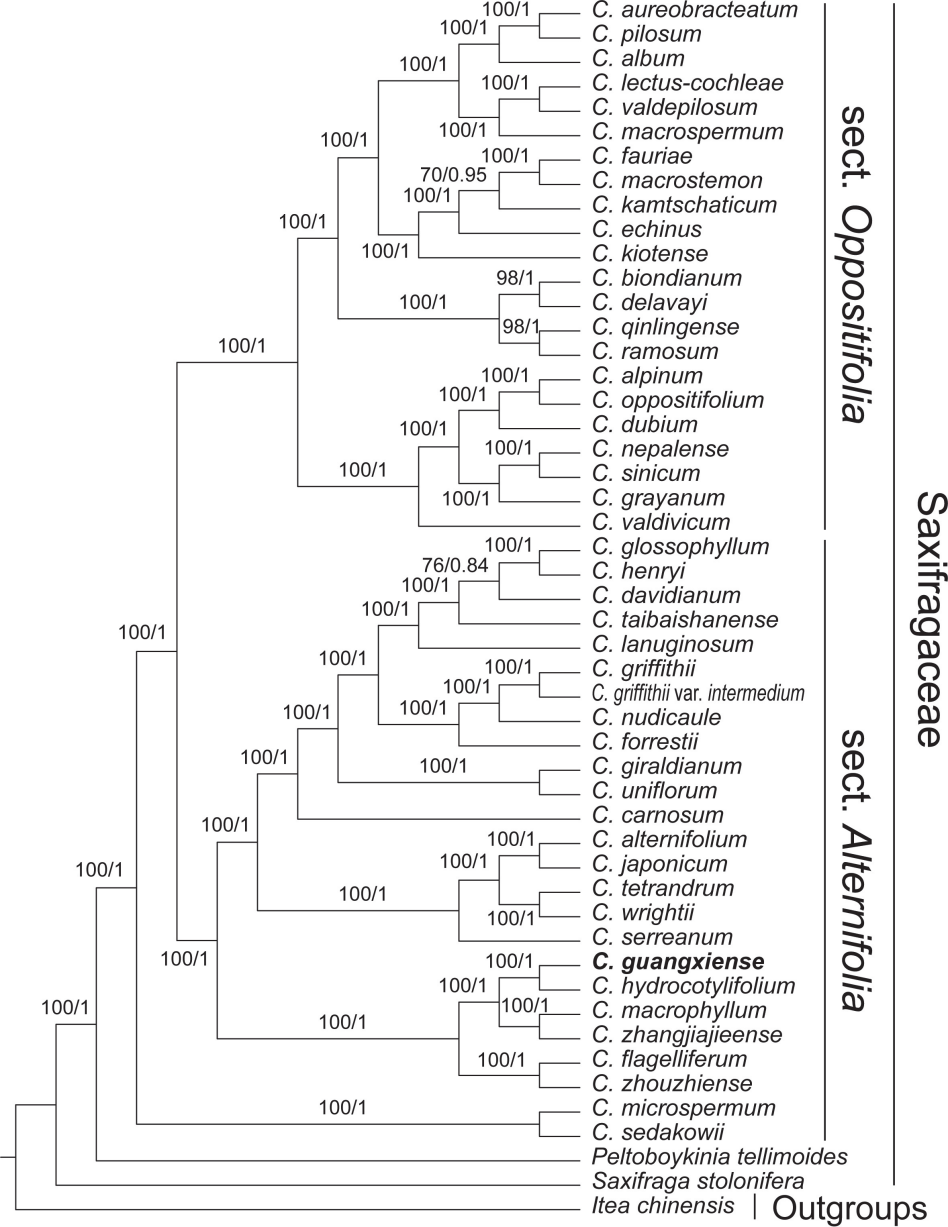
Phylogenetic tree of *Chrysosplenium* generated from maximum likelihood (ML) and Bayesian inference of cpPCGs+nrDNA dataset. Numbers below the branches indicate bootstrap values (≥50%) of the ML analyses and the posterior probability (≥0.5) of Bayesian analyses.

The cpPCGs matrix length was 71,919 bp, including 6,392 parsimony informative sites, 13,645 variable sites, and 55,865 conserved sites. The nrDNA matrix was 6,738 bp in length, with 765 parsimony informative sites, 1,200 variable sites, and 5,231 conserved sites. The cpPCGs+nrDNA matrix was 78,657 bp in length, with 7,157 parsimony informative sites, 14,845 variable sites, and 61,096 conserved sites. The phylogenetic tree of the cpPCGs matrix exhibited high confidence, while the phylogenetic tree of the nrDNA matrix had some branches with low support, and was significantly different from the former (Suppl. materials 1, 2). However, *Chrysospleniumguangxiense* was consistently related to *C.hydrocotylifolium* H.Lév. & Vaniot in both chloroplast and nuclear gene trees (Suppl. materials 1, 2). The phylogenetic tree of the cpPCGs+nrDNA matrix received a higher confidence value compared to trees generated from subsets (cpPCGs and nrDNA). Topologies obtained from BI and ML methods were congruent and showed that *Chrysosplenium* species clustered in a strongly supported clade (BS = 100%, PP = 1) which was further divided into three well-supported clades (defined as A-C clades; Fig. 2). *Chrysospleniumguangxiense* was recognized as a member of clade B and fell in its basal clade (BS = 100%, PP = 1; Fig. 2), which also included *C.macrophyllum* Oliv., *C.zhangjiajieense* X.L.Yu, Hui Zhou & D.S.Zhou, *C.hydrocotylifolium*, *C.flagelliferum* F.Schmidt, and *C.zhouzhiense* Hong Liu. Of these, *C.guangxiense* was most closely related to *C.hydrocotylifolium* (BS = 100%, PP = 1; Fig. 2). Although *C.glossophyllum* was also a member of clade B, it fell into a much more distant clade from *C.guangxiense* (Fig. 2).

### ﻿Morphological observations

A suite of morphological characters including rhizome size, leaf shape, leaf margin dentate, sepal apex shape, and seed size of *Chrysospleniumguangxiense* and *C.glossophyllum* was consulted or observed. *Chrysospleniumguangxiense* had a robust rhizome, basal leaves with a long cuneate base and fewer teeth in the margin, curled sepal margins, and red, larger seeds that make it easily distinguished from *C.glossophyllum* (Table 2). Considering the phylogenetic results, a morphological comparison between *C.guangxiense* and *C.hydrocotylifolium* was also conducted. *Chrysospleniumguangxiense* had long elliptic leaves and a long cuneate leaf base, which can be easily distinguished from *C.hydrocotylifolium* (Table 3).

**Table 2.** Diagnostic comparison of *Chrysospleniumguangxiense* and *C.glossophyllum*.

Characters	*C.guangxiense*	*C.glossophyllum*
Rhizome	Rhizome thick, crossed and nodular	absent
Basal leaves	base long cuneate, margin 10–20-crenate	base rounded to subcordate; margin 20–36-crenate
Sepals	margin curl	margin uncurl
Seed	red, 0.59–0.85 × 0.48–0.63 mm	black, 0.50 × 0.40 mm

**Table 3.** Diagnostic comparison of *Chrysospleniumguangxiense* and *C.hydrocotylifolium*.

Characters	*C.guangxiense*	*C.hydrocotylifolium*
Basal leaves and cauline leaves	Isophyllous	heterophyllous
Basal leaves	long elliptic, margin 14–24-crenate; base long cuneate	orbicular, margin 34–39-crenate; base reniform

**Table 4.** Summary of the complete plastid genome of *Chrysospleniumguangxiense*.

Characteristic	*Chrysospleniumguangxiense*
Size (base pair, bp)	154,004
LSC length (bp)	80,646
SSC length (bp)	17,118
IR length (bp)	28,120
Number of genes	111
Protein-coding genes	78
rRNA genes	4
tRNA genes	29
GC content	37.51%

**Table 5.** The gene statistics of the plastid genome of *Chrysospleniumguangxiense*. [Genes with one or two introns are indicated by one (^*^) or two asterisks (^**^), respectively. Genes in the IR regions are followed by the (×2) symbol].

Group of Genes	Gene Name	Number
tRNA genes	*trnK*-UUU, *trnQ*-UUG, *trnS*-GCU, *trnG*-GCC, *trnR*-UCU, *trnC*-GCA, *trnD*-GUC, *trnY*-GUA, *trnE*-UUC, *trnT*-GGU, *trnS*-UGA, *trnS*-CGA, *trnfM*-CAU, *trnS*-GGA, *trnT*-UGU, *trnL*-UAA*, *trnF*-GAA, *trnV*-UAC*, *trnM*-CAU, *trnW*-CCA, *trnP*-UGG, *trnR*-ACG(×2), *trnN*-GUU(×2), *trnI*-GAU*(×2), *trnA*-UGC*(×2), *trnL*-UAG, *trnI*-CAU(×2), *trnL*-CAA(×2), *trnV*-GAC(×2)	29
rRNA genes	*rrn16* (×2), *rrn23* (×2), *rrn4.5* (×2), *rrn5* (×2)	4
Ribosomal small subunit	*rpsl6*, *rps2*, *rps14*, *rps4*, *rps18*, *rps12* (×2), *rps11, rps8*, *rps3* (×2), *rps19* (×2), *rps7* (×2), *rps15*	12
Ribosomal Large subunit	*rpl33*, *rpl20*, *rpl36*, *rpl14*, *rpl16*, *rpl22* (×2), *rpl2* (×2), *rpl23* (×2)	8
DNA-dependent RNA polymerase	*rpoC2*, rpoC1*, *rpoB*, *rpoA*	4
Photosystem Ⅰ	*psaB*, *psaA*, *psaI*, *psaJ*, *psaC*	5
Large subunit of rubisco	*rbcL*	1
Photosystem Ⅱ	*psbA*, *psbK*, *psbI*, *psbM*, *psbD*, *psbC*, *psbZ*, *psbJ*, *psbL*, *psbF*, *psbE*, *psbB*, *psbT*, *psbN*, *psbH*	15
NADH dehydrogenase	*ndhJ*, *ndhK*, *ndhC*, ndhB*(×2), *ndhF*, *ndhD*, *ndhE*, *ndhG*, *ndhI*, ndhA*, *ndhH*	11
Cytochrome b/f complex	*petN*, *petA*, *petL*, *petG*, *petB*, *petD*	6
ATP synthase	*atpA*, atpF*, *atpH*, *atpI*, *atpE*, *atpB*	6
Maturase	*matK*	1
Subunit of acetyl-CoA carboxylase	accD*	1
Envelope membrane protein	*cemA*	1
Protease	clpP**	1
Translational initiation factor	*infA*	1
c-type cytochrome synthesis	*ccsA*	1
Conserved open reading frames(*ycf*)	ycf3**, *ycf4*, *ycf2* (×2), *ycf1* (×2)	4

## ﻿Discussion

Our phylogenetic result supported the monophyly of *Chrysosplenium* (Soltis et al. 2001; Fu et al. 2021; Yang et al. 2023). Besides two well-defined clades (denoted as sect. Oppositifolia and sect. Alternifolia), our result also revealed a third clade comprising two species from sect. Alternifolia, the topology of which is consistent with the previous study (Yang et al. 2023). This phylogenetic relationship indicated a non-monophyletic status of sect. Alternifolia and suggested that a deeper morphological character evolution across this phylogenetic framework is needed to evaluate the phylogenetic informativeness of characters.

**Figure 3.**
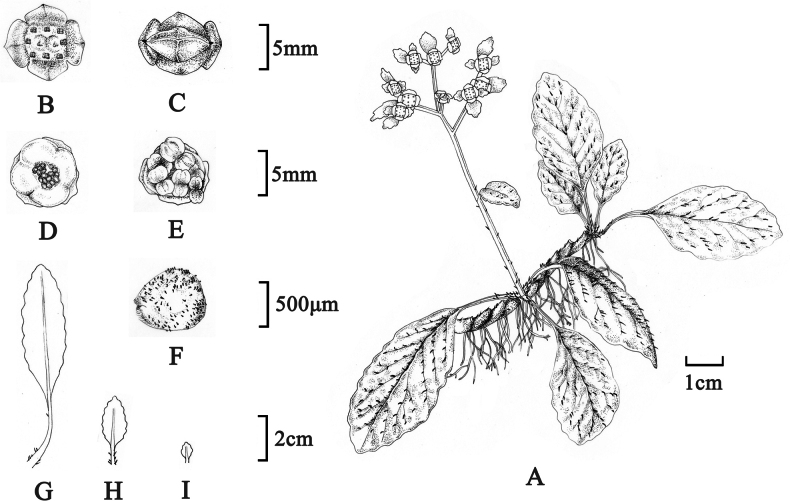
Illustration of *Chrysospleniumguangxiense* H.G.Ye & Gui C.Zhang **A** habit in flowering phase **B** flower **C** indehiscent capsule **D** dehiscent capsule and seeds **E** germinated seeds in capsule **F** seeds **G** caulline leaf **H, I** bracteal leaf.3

**Figure 4.**
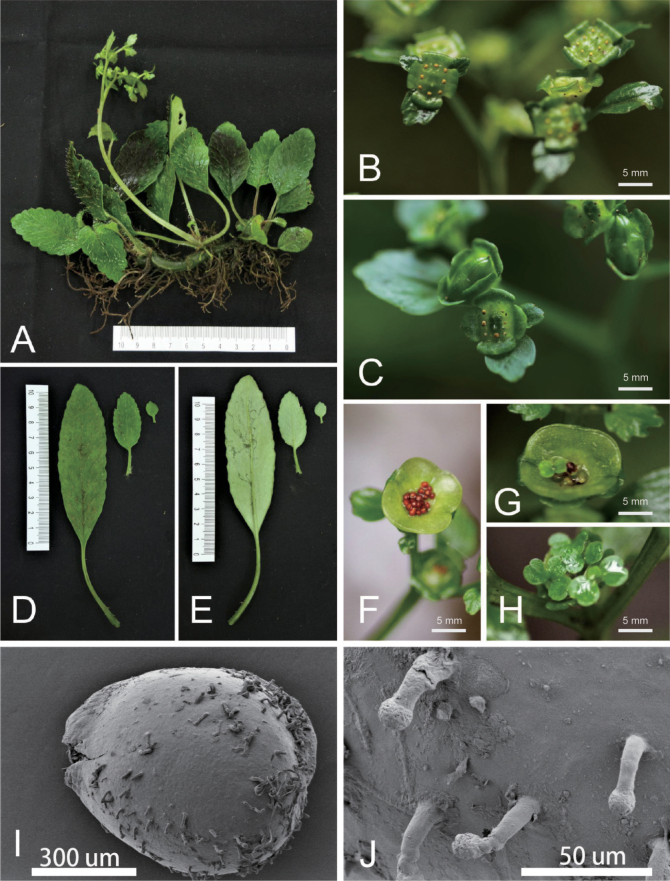
Plate of *Chrysospleniumguangxiense* H.G.Ye & Gui C.Zhang **A** habit **B, C** inflorescence with flowers **D, E** basal leaves **F** fruit and seed **G, H** germinated seeds and seedlings in capsule **I, J**SEM of seed (Photos by Hong Liu).

In our phylogenetic tree, *Chrysospleniumguangxiense* was recovered as a member of sect. Alternifolia, most closely related to *Chrysospleniumhydrocotylifolium* (BS = 100%, PP = 1) but had a distant relationship with *C.glossophyllum*. It was easy to distinguish *C.guangxiense* from *C.hydrocotylifolium* by the long elliptic leaves and long cuneate leaf bases (Table 3). Our morphological comparison between *C.guangxiense* and *C.glossophyllum* also showed a suite of characters, including having a robust rhizome, basal leaves with a long cuneate base and fewer teeth in margin, and larger seeds in *C.guangxiense* which helped distinguish it from *C.glossophyllum* (Table 2). Furthermore, there was a typical viviparous phenomenon of *C.guangxiense*; the mature seeds were able to germinate directly in the opening capsule (Figs 3E, 4G, H). This feature has not been reported in any other *Chrysosplenium* species so far. Therefore, our molecular and morphological evidence supports *C.guangxiense* as a distinct species that resurrected from *C.glossophyllum*. We presented the following detailed taxonomic treatment for *C.guangxiense*.

### ﻿Taxonomic treatment

#### ﻿ Chrysosplenium guangxiense

Taxon classificationPlantaeSaxifragalesSaxifragaceae

H.G.Ye & Gui C.Zhang in Acta Bot. Austro Sin. 9: 57, f. 1 (1994)

5F00CD5C-24B3-5D92-AB92-0C2B3AD0555D

Figs 3 , 4

##### Type.

Tian Lin, 11 Oct. 1989, South China Exped. 2458 (holotype: IBSC!; isotype: IBSC!).

##### Description.

Perennial herbs, 5.5–17 cm high. ***Root*** fibrous and robust. Long creeping ***rhizome*** developed, thick, crossed and nodular, 1–2 cm between each node, without stolons and bulbs. ***Flowering stem(s)*** always 1, erect, branched, 10–17 cm high, sparsely pilose, green, squared. ***Sterile branches*** arise from all basal leaves. ***Isophyllous, Basal leaves*** 3–6, alternate and clustered; petiole 1–6.5 cm long, white pilose; leaf blade long elliptic, 2.2–10.3 × 1.8–3.3 cm, abaxially glabrous, light green, adaxially with sparse long hispid, dark green, apex rounded, margin 14–25-crenate, base long cuneate. ***Cauline leaves*** always 1, petiole 1.3–2.2 cm long; blade 2.2–4.0 × 1.2–1.9 cm, long elliptic, glabrous in the abaxial side and with sparse hispid in the adaxial side; apex obtuse; margin obtusely dentate (9–13 teeth); base broadly cuneate; veins obvious in adaxial. ***Pleiochasium*** 9–16 cm wide, 10–15 cm high, extremely diffused, with 5–20-flowered cyme, branches sparsely hispid, surrounded by bracts; ***bracteal leaves*** green, elliptic to broadly ovate or round, glabrous. ***Flowers*** tetramerous, actinomorphic; ***sepals*** 4 (2 pairs), flat, green, 0.9–1.2 × 2.1–4.3 mm, broadly ovate, apex acute, with margin curls outward in fruiting time; disk obvious; ***stamens*** 8, ca. 0.2 mm long, shorter than sepals; anthers orange, 2-locular, longitudinally dehiscent; ovary 2-locular, semi-inferior; stigma 2; styles erect, ca. 0.2 mm long. ***Fruit*** a capsule, 5–7 mm long, green, smooth, 2-lobed (horn-shaped), equal, dehiscent along the adaxial suture; seeds numerous, red or reddish brown, obovoid, a raphe on one side, 594.19–855.33 × 475.41–625.7 μm, long papillose. Viviparous.

##### Additional specimen examined.

*Chrysospleniumglossophyllum*. China. Sichuan: Kuan County (Dujiangyan City), 19 April 1930, F. T. Wang 20553 (PE!, NAS!); same locality, 6 May 1987, Xintang Ma & Zhilong Zhao 87-0521 (WCSBG!); same locality, 15 April 2013, LiXJ 353 (KUN!); same locality, 24 May 2016, Hong Liu, HSN06644 (HSN!); same locality, 26 October 2017, Hong Liu, HSN08105 (HSN!). *Chrysospleniumguangxiense*. China. Guangxi: Lingyun County, Baise City, 6 March 2014, Lingyun team 451027140305005 (GXMG!); Tianlin County, Baise City, 27 November 2019, Hong Liu HSN13356 (HSN!).

##### Conservation status.

*Chrysospleniumguangxiense* is only known from two localities (IUCN criterion D2). At these two localities, the populations included ca. 200 mature individuals (IUCN criterion D1) growing in several patches. Using the IUCN methodology, *C.guangxiense* is classified as Vulnerable (VU) based on criteria D1 and D2: population size and the number of locations, combined with a plausible future threat that could drive this taxon to Critically Endangered or Extinct in a very short time. However, the vivipary of *C.guangxiense* may strengthen its adaptability to cope with future climate and environmental changes. The future threat is mainly due to grazing.

## ﻿Conclusions

The phylogenetic analyses using plastomes and nuclear gene sequences of *Chrysospleniumguangxiense* reveal that *C.guangxiense* belongs to the sect. Alternifolia, is closely related to *Chrysospleniumhydrocotylifolium*, but distant from *C.glossophyllum* based on leaf morphology and other traits. Our findings support the resurrection of *C.guangxiense* as a distinct species and provide a detailed taxonomic treatment for its identification. The phylogenetic analyses confirm the monophyly of *Chrysosplenium* and reveal a non-monophyletic status of sect. Alternifolia. Further systematic studies of *Chrysosplenium* should focus on finding additional morphological characters with phylogenetic informativeness to disentangle the non-monophyletic sect. Alternifolia, and propose a new infrageneric classification and provide a stable framework for answering broader questions in evolutionary biology.

## Supplementary Material

XML Treatment for Chrysosplenium guangxiense

## ﻿Additional information

### Author ORCIDs

Long-Fei Fu https://orcid.org/0000-0001-8708-4718

Tian-Ge Yang https://orcid.org/0000-0003-1681-1767

De-Qing Lan https://orcid.org/0000-0003-3277-357X

Xi-Tang Chen https://orcid.org/0000-0002-5461-7183

Hong Liu https://orcid.org/0000-0001-7227-2476

### Data availability

All of the data that support the findings of this study are available in the main text or Supplementary Information.

## Supplementary materials

Supplementary material 1 Phylogenetic tree of *Chrysosplenium* generated from maximum likelihood (ML) of cpPCGs dataset Data type jpg Explanation note Numbers on the branches indicate bootstrap values (left) of the ML analyses and the posterior probability (right) of Bayesian analyses. https://binary.pensoft.net/file/1078978This dataset is made available under the Open Database License (http://opendatacommons.org/licenses/odbl/1.0/). The Open Database License (ODbL) is a license agreement intended to allow users to freely share, modify, and use this Dataset while maintaining this same freedom for others, provided that the original source and author(s) are credited.Long-Fei Fu, Tian-Ge Yang, De-Qing Lan, Xi-Tang Chen, Hong Liu

Supplementary material 2 Phylogenetic tree of *Chrysosplenium* generated from maximum likelihood (ML) of nrDNA dataset Data type jpg Explanation note Numbers on the branches indicate bootstrap values (left) of the ML analyses and the posterior probability (right) of Bayesian analyses. https://binary.pensoft.net/file/1078979This dataset is made available under the Open Database License (http://opendatacommons.org/licenses/odbl/1.0/). The Open Database License (ODbL) is a license agreement intended to allow users to freely share, modify, and use this Dataset while maintaining this same freedom for others, provided that the original source and author(s) are credited.Long-Fei Fu, Tian-Ge Yang, De-Qing Lan, Xi-Tang Chen, Hong Liu
