# Supplementary figures and images for: ﻿Molecular and morphological evidence supports the resurrection of Chrysospleniumguangxiense H.G.Ye & Gui C.Zhang (Saxifragaceae)
Source: PhytoKeys. 2024 Jun 25;243:185–98. doi: 10.3897/phytokeys.243.125742 (PMC11220401; doi:10.3897/phytokeys.243.125742)

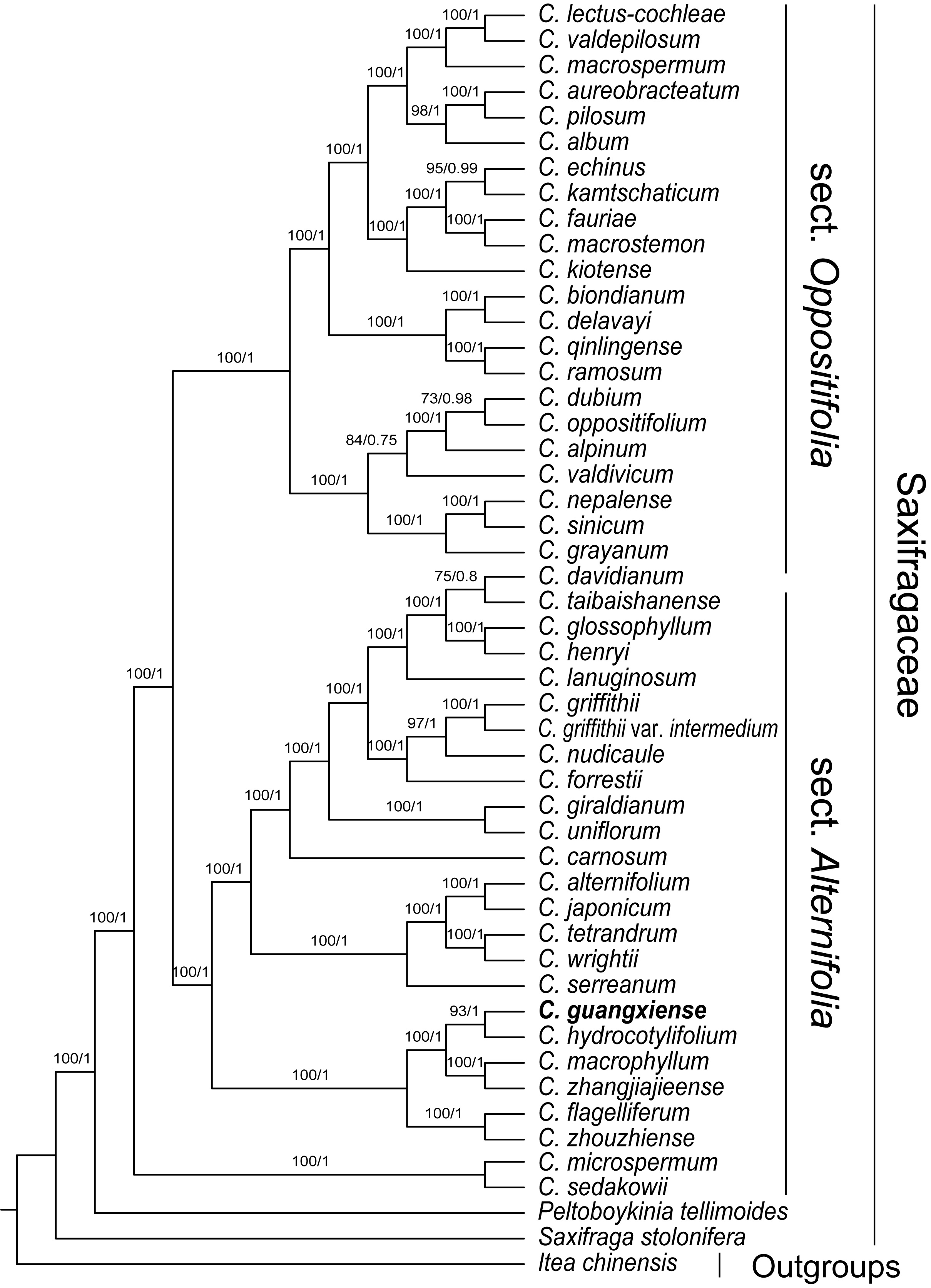

Supplement: Supplementary material 1 — Phylogenetic tree of Chrysosplenium generated from maximum likelihood (ML) of cpPCGs dataset [file phytokeys-243-185_article-125742__-s001.jpg]

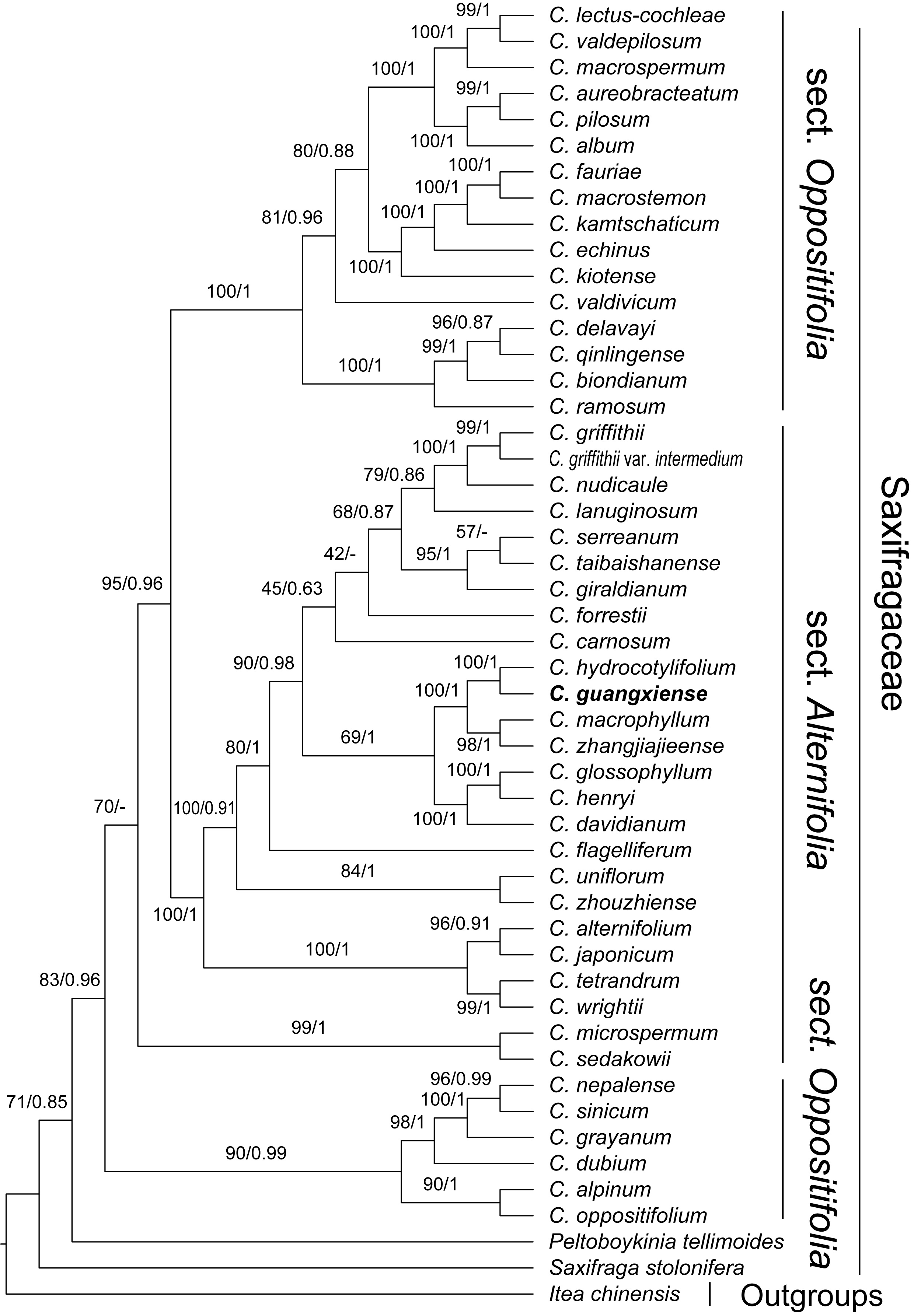

Supplement: Supplementary material 2 — Phylogenetic tree of Chrysosplenium generated from maximum likelihood (ML) of nrDNA dataset [file phytokeys-243-185_article-125742__-s002.jpg]
